# Telepathology consultation in China using whole slide image and an internet based platform

**DOI:** 10.1186/1746-1596-8-S1-S10

**Published:** 2013-09-30

**Authors:** Chen Zhou, Amir Rahemtulla, Liu Tong Hua, Huaqiang Shi

**Affiliations:** 1 Department of Pathology, University of British Columbia, Vancouver, Canada; 2 Department of Pathology, Peking Union Medical College , Beijing, China; 3 Department of Pathology, General Hospital of Air Force, Beijing, China

## Background

Telepathology is a particularly useful pathological tool suitable for developing countries such as China, which generates a large number of pathology specimens each year due to the size of its population but has a shortage of well trained and experienced pathologists in primary cares hospitals and hospitals within under-developed regions. Pathologists in these hospitals often have difficulty diagnosing challenging pathology cases. Telepathology, especially second opinion or teleconsultation is one of the solutions to this problem [1-3]. We reported an internet based open telepathology consultation platform using whole slide images (WSI) in China. The results from the telepathology consultation service since inception have been analyzed and summarized for this publication. We believe that our experiences will help to promote telepathology in China and in other developing countries.

## Methods

The telepathology consultation cases used in this report represent pathology consultation sent to the telepathology consultation platform (http://www.mpathology.cn/mpcc/) from the beginning of the service in July 2008 to May 30, 2011. The cases submitted for teleconsultation were from 29 institutions, which were equipped with a virtual microscope, Motic Virtual Microscopic Scanner (Motic, China). The equipment and related software used in this report were validated in a previous telepathology study using a variety of 600 surgical pathology specimens [4]. When the participating hospital had a pathology case requiring consultation, a referring pathologist logged into the website, http://www.mpathology.cn/mpcc/ with a secure user name and password and filled out an online request form which included the patient’s name, age, and relevant clinical information, gross findings, immunohistochemistry results, a preliminary diagnosis and the name of expert pathologist chosen for consultation. Referring pathologist then scanned, uploaded and attached WSI of one or several representative H/E slides as well as relevant immunohistochemistry stained slides to the request form, and then sent these to the platform.

An internet based telepathology platform was used and a server was used for storage of images and data. The system was maintained by two information technology (IT) technicians and one system manager. When referring pathologists from submitting hospital sent request for consults to the platform, IT technicians would be alerted by e-mails. The technician would then exam the completeness of submitted materials and system manager would contact the expert pathologist for consultation.

A panel of 84 Chinese pathology experts was invited and agreed to participate in the teleconsultation service. The names, affiliation of the pathologists and the areas of their subspecialty expertise were listed on the website (http://www.mpathology.cn/mpcc/). The platform covered the medical malpractice insurance for the expert consultants. When an expert pathologist was requested for consultation, the expert was instantly noticed by the cell phone message and e-mail. The expert pathologist would use a computer or an Ipad to log into the website, review WSI, capture a representative image, write the pathologic diagnosis, sign the report with an electronic signature and then release the final report.

Once the final report was released, the system sent an email to alert the system manager who then sent the final consultation report by e-mail or fax to the referring pathologist.

Statistical analysis was performed using SPSS 7.0 edition.

## Results

### Consultation cases submitted by hospitals

The number of cases submitted increased from 17 cases in 2008 to 587 cases in 2010. 935/1022 cases (91%) were sent in 2010 and the first 5 months of 2011.

A total of 29 hospitals were participated in the telepathology consultation service. The number of participating hospitals was 3 in 2008, 9 in 2009, and 21 in 2010; a 7-folds increase from 2008 to 2010. The mean number of cases submitted by each hospital was 35 cases in the past 3 years. Ten hospitals accounted for 90% of the cases submitted, with the other 19 hospitals accounting for only 10% of the submitted cases.

The average number of WSI per case was 1.8 with a range from 1 to 11. 62.0% of the cases only had one WSI, 27.6% had two WSI, and 10.4% had 3 or more WSI. 203 (19.8%) of the cases had included immunohistochemistry slides.

### Turnaround time

The average time needed from transmitting teleconsultation request form with attached WSI image to the server to the release of teleconsultation pathology report by expert consultants was 38 hours with a range of less than 1 hour to 323 hours. The teleconsultation report was released within 12 hours for 43.2% of cases; 24 hours for 65.9% of cases and 48 hours for 79% of cases (Table 1).

**Table 1:** Turnaround time for teleconsultation

Time (hours)	No. Cases	Percent(%)
**<12**	441	43.2

**12-24**	232	22.7

**24-48**	134	13.1

**48-168**	151	14.8

**>168**	64	6.2

### Expert consultants

Although a list of 84 expert consultants was available for teleconsultation, only 43 expert consultants were requested by submitting hospitals for teleconsultation. Among these 43 experts, 23 consultants were requested most frequently, accounting for 95% of the consultation cases.

### Pathology site of the submitting cases

The common sites of the pathology were gynecologic (24.2%), gastrointestinal/ liver/pancreatic (14.7%), and lung (13.1%), each accounting for more than 10% of cases. 810 (79.3%) out of 1022 were neoplastic pathology. Among them, 193 (21.3%) were benign tumors but 637 (78.7%) were malignant.

### Agreement of second opinion with preliminary diagnosis provided by referring pathologists

302 (29.5%) out of 1022 cases were not given a preliminary pathology diagnosis by submitting hospitals. Among 720 cases with a preliminary pathology diagnosis, 122 (16.9%) of the cases received a consultation report which was not in agreement with the preliminary pathology diagnosis (Table 2). Local hospital pathologists could not render a preliminary diagnosis or made a wrong preliminary diagnosis in 424 cases, representing 41.5% of the total teleconsultation cases.

**Table 2:** Agreement of expert opinions with preliminary diagnosis.

Preliminary diagnosis	No. Cases	Agreement of expert opinions with preliminary diagnosis			
		
		Concordance	General concordance	No concordance	No explanation
**Yes**	720	282	218	122	98

**No**	302	-	-	-	-

**Total**	1022	282	219	122	98

## Discussion

Our results indicated that telepathology consultation in China has gained traction since 2010. More than 90% of consultation cases submitted during the 3 years of this study were in 2010 and the first 5 months of 2011. The main reason is the governmental support for telepathology consultations. In 2010, the Ministry of Health in China released a document encouraging the use of telepathology across the country; other reasons are the availabilities of high speed internet and 3G networks and low cost commercial virtual microscopes in China after 2009.

Ideally, it is would be best to submit WSI of all tissue slides to the expert pathologist for teleconsultation as in traditional pathology consults. However, submitting and viewing all digitalized WSI from a single case is time consuming and more expensive. In this report, about 90% cases had ≤2 WSI. Our report indicates that using WSI in teleconsultation is effective and that most cases need only 1 or 2 WSI.

An internet based open platform used in this study has several advantages. First, expert pathologists are from multiple institutions and enjoy national reputations in their subspecialties. Second, the platform can be accessed by expert pathologists at anytime or places where internet or a Wi-Fi connection is available. Third, a centralized open platform can accept a large number of consultation cases, creating an economic model for the long-term survival. In several previous reports, the telepathology consultation services were either closed systems or offered only local service [5,6]. Such systems often had limited cases and were economically not viable.

One of the advantages of teleconsultation is a much shorter turnaround time(TAT), as compared with a mean of 6 days TAT in traditional pathology consultation [7]. Our results show that the average TAT was 38 hours and that about 2/3 of cases had a TAT within 24 hours. Approximately 10% of cases had a TAT longer than 7 days, this is due to expert pathologists requested additional slides or immunohistochemistry staining for some cases before rendering a final diagnosis.

Our analysis showed that although 84 expert consultants have been listed on the website, only 43 of them had participated in the consultation service and 23 of them completed 90% of the consultation work. The result indicated that a large telepathology consultation service may operate using only about 25 pathologists with different subspecialty expertise, reducing the cost of malpractice insurance and the difficulty for referring pathologists to select an expert pathologist.

In this report, we found that gynecologic specimens, gastrointestinal and live/pancreatic specimens and lung specimens were the most common specimens sent for consultation. A high proportion of head and neck, soft tissue, and hematopoietic pathology cases were also among the more frequent consultation specimens. Unlike consultation cases in Western countries, a low proportion of cases consisted of skin pathology [8] . This is similar to the findings of a previous telepathology study in China [4].

In our study, about 30% of cases did not come with a preliminary pathology diagnosis. This may be related to the degree of difficulty of those submitted cases, and/or due to the lack of pathology training and experience of submitting pathologists in China. Among those having preliminary pathology diagnosis, 16.9% were not in agreement with the expert diagnosis. This is in agreement with previous reports [9,10].

Our analysis showed that consultation cases without preliminary diagnosis and with the wrong preliminary diagnosis accounted for 41.5% of teleconsultation cases. This indicated pathologic diagnosis was valuable and clinically significant in 40% of the teleconsultation cases. This finding demonstrated that telepathology has an important and practical role in pathology consultation in China.

## Competing interests

None

## Authors’ contributions

CZ: participating the study, analysis and summary of the data and writing up manuscript

AR: direct analysis and writing up manuscript

LTH: directing the study and reviewing the manuscript

HS, participating and supervising the study, and reviewing manuscript
